# Induced triple clutching in captivity for three falcon species: Common Kestrel (*Falco tinnunculus*), Saker Falcon (*Falco cherrug*) and Peregrine Falcon (*Falco peregrinus*)

**DOI:** 10.3897/BDJ.14.e197934

**Published:** 2026-08-24

**Authors:** Rusko Petrov, Alian Valchev

**Affiliations:** 1 Trakia University, Stara Zagora, Bulgaria Trakia University Stara Zagora Bulgaria; 2 Green Balkans - Stara Zagora NGO, Stara Zagora, Bulgaria Green Balkans - Stara Zagora NGO Stara Zagora Bulgaria; 3 Vet Centre Stara Zagora, Stara Zagora, Bulgaria Vet Centre Stara Zagora Stara Zagora Bulgaria

**Keywords:** breeding management, chick survival, egg production, *Falco cherrug*, *Falco peregrinus*, *Falco tinnunculus*, falcons, hatching success, increasing productivity of genetically valuable breeding pairs, raptor conservation, reproductive biology

## Abstract

Triple clutching within a single breeding season is rarely documented in falcons, particularly under captive conditions. This study reports the occurrence of triple clutching in three falcon species — Common Kestrel (*Falco tinnunculus*), Saker Falcon (*Falco cherrug*) and Peregrine Falcon (*Falco peregrinus*) — reared in captivity in Bulgaria. Two breeding pairs per species were monitored during the 2011–2020 breeding seasons under controlled conditions with optimal husbandry and nutritional regimes. Clutch frequency, egg production, fertility rates, hatching success and chick survival were recorded for each pair. Results revealed that both monitored pairs of each species produced three successive clutches within a single season, with varying degrees of reproductive success. These findings suggest that under good captive conditions, triple clutching is a feasible reproductive strategy in falcons and may significantly enhance the productivity of conservation breeding programmes. Further research is needed to determine the physiological limits and welfare implications of induced high‑frequency breeding in raptors

Triple clutching within a single breeding season is rarely documented in falcons, particularly under captive conditions. This study reports the occurrence of triple clutching in three falcon species — the Common Kestrel, Saker Falcon and Peregrine Falcon — maintained in captivity and monitored over the 2011–2020 breeding seasons. Two breeding pairs per species were observed under controlled husbandry and nutritional conditions and clutch frequency, egg production, fertilisation, hatching success, chick survival and breeding outcome were recorded for each pair.

All monitored pairs produced three successive clutches within a single breeding season, although the mechanism differed amongst species. Triple clutching occurred naturally in Common Kestrels under full parental care, whereas in Saker and Peregrine Falcons, it was induced through egg removal and artificial incubation. Reproductive output remained high across successive clutches, with viable eggs and healthy chicks produced in all three species, despite some reduction in egg size in later clutches of Saker and Peregrine Falcons.

These findings demonstrate considerable reproductive plasticity in falcons and show that, under favourable captive conditions, triple clutching can substantially increase breeding output. The results highlight the practical value of multi-clutch breeding for captive propagation and conservation programmes involving re-introduction or population reinforcement.

## Introduction

Falcons, comprising nearly 60 species within the family Falconidae (order Falconiformes), are diurnal raptors distinguished by their elongated, pointed wings and robust, high-speed flight. The term “falcon” is often used specifically to refer to members of the genus *Falco*, which includes over 35 globally distributed species. These birds exhibit considerable size variation, ranging from approximately 15 cm in the smallest species (e.g. falconets of the genus *Microhierax*) to about 60 cm in the gyrfalcon (*Falco rusticolus*), the largest species native to Arctic regions. This variation is also reflected in the three species examined in the present study: the Common Kestrel (*Falco tinnunculus*) is a small and slender falcon, the Saker Falcon (*Falco cherrug*) is a larger and more robust species and the Peregrine Falcon (*Falco peregrinus*) is a medium-to-large, compact and powerful falcon. In *Falco* species, females are typically larger and more aggressive than males, a trait that makes them more suitable for traditional falconry practices (Britannica 2026).

Falconids are predominantly monogamous, with polygyny rarely observed and cooperative breeding regularly documented in Ibycter americanus and Microhierax caerulescens (Snyder 2001). Most species breed as solitary territorial pairs, although some nest colonially under certain conditions (Kemp and Newton 2003). In migratory taxa, males typically arrive at breeding sites before females and initiate courtship displays, which may be performed alone or in pairs (Snyder 2001). Amongst the three species examined here, the Common Kestrel is known to adapt readily to captivity and may breed successfully even in communal aviaries, whereas the Saker Falcon and the Peregrine Falcon are more commonly maintained as separate breeding pairs under managed conditions.

Parental roles are strongly divided: females brood, feed and defend the young, while males provide prey until females begin hunting mid-way through the nesting period (Kemp and Newton 2003). Breeding occurs once annually, generally during peak prey availability between late winter and early summer. Clutch size ranges from 1–7 eggs (most commonly 2–4), which are laid at 2–3 day intervals. In the three species examined in the present study, it is usually around 4–6 eggs in the Common Kestrel and Saker Falcon and 3–5 eggs in the Peregrine Falcon. Eggs are incubated solely by the female for 28–35 days, with hatching usually synchronous, in contrast to Accipitridae, where asynchrony and siblicide are common. Fledging occurs after approximately 28–30 days in the Common Kestrel, whereas in the larger Saker Falcon and Peregrine Falcon, the nesting period is typically longer and may extend beyond one month. Juveniles remain dependent on parental provisioning for two weeks to several months post-fledging (Snyder 2001, Kemp and Newton 2003).

Most species attain reproductive maturity at 1–3 years of age and are typically philopatric, returning to natal areas to breed. Nesting preferences amongst the three species examined in the present study also vary, with the Common Kestrel readily using cavities and nest boxes (E et al. 2019), the Saker Falcon often breeding on artificial nests or platforms in open landscapes (Rahman et al. 2014) and the Peregrine Falcon typically nesting on cliffs or on tall buildings in urban environments (Brambilla et al. 2006, Mak et al. 2021).

## Material and Methods

Throughout 2011–2020, we studied two pairs each of Common Kestrels, Saker Falcons and Peregrine Falcons in a controlled setting. Common Kestrels were studied during the 2011–2012 breeding seasons at the Wildlife Rehabilitation and Breeding Centre, operated by Green Balkans - Stara Zagora NGO, Bulgaria; Saker Falcons during the 2015–2016 breeding seasons at the Breeding Centre for Birds of Prey, Burgas, Bulgaria; and Peregrine Falcons during the 2019–2020 breeding seasons at the Centre for Birds of Prey, Bucharest, Romania. The enclosures were specifically designed to cater to their reproductive and behavioural requirements. Birds were housed either in communal or individual breeding aviaries designed according to species‑specific biological and behavioural requirements. Common Kestrels were housed in aviaries measuring 3.0 × 2.5 × 2.5 m (length × width × height) and provided with enclosed nest boxes measuring 40 × 30 × 30 cm, with a single frontal opening. Saker Falcons and Peregrine Falcons were maintained in breeding aviaries measuring 5.0 × 3.0 × 3.0 m and provided with nest boxes measuring 100 × 50 × 50 cm, featuring two open sides to facilitate access and observation. All enclosures provided sufficient space for flight, elevated perches, appropriate nesting structures, visual barriers to reduce disturbance and exposure to natural photoperiod and ambient climatic conditions. Sheltered sections were available in all aviaries to protect from adverse weather (Petrov et al. 2022). Breeding behaviour was monitored using Dahua IP security cameras with 2–8 megapixel resolution, which recorded movement continuously to NVR devices. This system allowed 24-hour observation of laying, incubation, hatching, parental behaviour and disturbance events, while minimising direct human presence near the aviaries. Camera footage was also used for remote veterinary assessment by veterinarians specialised in raptors, particularly when evaluating behaviour, posture, feeding activity, signs of stress and potential medical problems.

All birds were classified as non-releasable due to behavioral alterations caused by prolonged human interaction or injuries that compromised their ability to survive in the wild. The Common Kestrel pairs formed naturally, as the aviary housed eight to ten individuals at all times. Although this species is typically territorial, there is substantial evidence that they can thrive and reproduce in communal settings (Kirkwood 1980). The other two species were kept in separate aviaries. The primary goal was to enhance breeding experience for these species and eventually re-introduce the chicks into the wild through hacking (Lazarova et al. 2021).

Our feeding protocols were informed by existing literature on the dietary composition of these raptor species. We implemented a two-week rotational feeding system designed to provide dietary variety, while maintaining a consistent supply of whole-prey items (Table 1, Table 2). Following the hatching of the first egg, we suspended the fasting day and increased rodent provision to meet the heightened energy requirements. Feeding occurred during the warmer hours of the day to align better with the birds' natural hunting behaviour (Rijnsdorp et al. 1981).

In Common Kestrels, all clutches were incubated naturally by the female without egg removal. In Saker and Peregrine Falcons, clutches were manipulated through systematic egg removal after the fourth or fifth egg was laid and removed eggs were artificially incubated to induce replacement clutch formation (Morrison and Walton 1980, Wood and Collopy 1993). Artificial incubation was carried out in a Falcon C-30SK incubator at 37.3°C and 10% relative humidity, with automatic egg turning throughout incubation. Egg fertilisation was assessed by candling between days 7 and 10 of incubation, when early embryonic development could be reliably detected. Chicks hatched through artificial incubation were kept under controlled conditions for 5–7 days after hatching to allow veterinary assessment by raptor-specialised veterinarians and to ensure adequate post-hatching stabilisation. Once clinically stable and sufficiently strengthened, they were transferred to suitable foster parents for further rearing whenever appropriate. For each clutch, the number of eggs laid, fertilisation rate, hatching success, chick survival and final fate of juveniles (released or retained within the breeding programme) were recorded.

Given the limited number of breeding pairs included in the present study, these findings should be interpreted cautiously and considered as evidence of reproductive potential under controlled captive conditions rather than as broadly generalisable species-level patterns. The study was conducted at officially authorised facilities for the keeping, rehabilitation and breeding of wild birds. The Centre for Birds of Prey, Bucharest, Romania, was officially registered as a livestock facility “Centre for Birds of Prey” under Order No. 9328 of 28 November 2013, issued by the Minister of Environment and Water. The Breeding Centre for Birds of Prey, Burgas, Bulgaria, was officially registered as a livestock facility “Centre for Birds of Prey” under Order RD 8017-0122 of 15 July 2013, issued by the Minister of Environment and Water. The Wildlife Rescue Centre of Green Balkans was officially designated as a Wildlife Rescue Centre under Order RD 619/08 September 2015 of the Minister of Environment and Water. The activities of these facilities are regulated by Chapters III and IV of the Biological Diversity Act and the Green Balkans Wildlife Rescue Centre also operates under the specialised Regulation on the activities of wildlife rescue centres, published in State Gazette No. 14 of 20 February 2004. All procedures involving birds were conducted in accordance with accepted standards for humane treatment, animal welfare and species-appropriate husbandry. Handling, veterinary assessment, incubation, chick care and breeding management were performed by responsible personnel with appropriate professional training, experience and certification for working with wild birds and raptors.

## Results

Induced triple clutching in the Common Kestrel was consistently observed under fully natural parental conditions, without egg removal or artificial incubation. Both monitored pairs completed three successive clutches within a single breeding season across two consecutive years (2011–2012). Initial laying occurred in late February (ca. 23 February) and females initiated subsequent clutches approximately 7–10 days after hatching of the previous brood, resulting in temporal overlap between chick rearing and egg-laying. The second clutch in both pairs was consistently lost due to rat predation 6–9 days after the last egg was laid; however, females resumed laying approximately 10 days after clutch failure, producing a third clutch in mid-April, with hatching occurring around 20 May. Clutch size remained stable (4–6 eggs) and productivity in successful clutches was consistent (4–5 fledglings), demonstrating a high capacity for rapid re-nesting and sustained reproductive effort under natural conditions (Table 3, Table 4).

In the Saker Falcon, triple clutching was induced through systematic egg removal and artificial incubation during the 2015–2016 breeding seasons in Burgas. Both pairs completed three successive clutches per season, with the first clutch initiated in early February (ca. 1 February). Eggs were removed after the fifth egg was laid, triggering re-laying approximately 20 days later. Subsequent clutches were recorded around 25 February and 20 March, respectively, indicating a consistent re-nesting interval under induced conditions. Clutch size remained stable (4–6 eggs), although a reduction in egg size was observed in later clutches, particularly after the 8^th^–10^th^ egg laid within a season. This reduction was most evident in the third clutch and may reflect the cumulative demands of repeated egg formation within a short breeding period. Nevertheless, the smaller egg size did not appear to negatively affect fertility, hatchability or chick survival and all chicks were released into the wild (Table 5, Table 6).

A similar induced pattern of triple clutching was observed in the Peregrine Falcon during the 2019–2020 breeding seasons in Bucharest. Two pairs completed three successive clutches per season under artificial incubation protocols. The first clutch was initiated in early February (ca. 6 February), with eggs removed after the fourth to fifth egg (ca. 12 February), followed by subsequent clutches around 4 March and 26 March. As in Saker Falcons, females resumed laying approximately 20 days after egg removal, demonstrating a predictable re-nesting interval. Clutch size remained consistent (4–5 eggs), although, as observed in Saker Falcons, later clutches produced smaller eggs after the 8^th^ egg within a season. The decrease in egg size was most apparent during the third clutch, suggesting a possible cumulative effect of repeated laying under induced breeding conditions. Nevertheless, no negative effect on embryo viability or chick survival was observed. In contrast to Saker Falcons, all juveniles from Peregrine Falcons were retained within the breeding programme and were not released into the wild (Table 7, Table 8).

## Discussion

The results of this study demonstrate that all three investigated falcon species are biologically capable of producing three clutches within a single breeding season under favourable conditions. However, the mechanisms underlying triple clutching differed markedly between species. In the Common Kestrel, triple clutching occurred without artificial intervention and under fully natural parental care, whereas in Peregrine and Saker Falcons, repeated clutch production was induced through systematic egg removal. These contrasting reproductive pathways highlight both the intrinsic reproductive plasticity of falcons and the strong influence of management practices on breeding output in captivity (Morrison and Walton 1980, Burnham et al. 2003).

### Natural versus induced triple clutching

Natural triple clutching observed in Common Kestrels closely resembles replacement and overlapping brooding strategies reported in small raptors. Females resumed egg laying, while still provisioning dependent young, indicating that egg formation can proceed concurrently with chick rearing under favourable energetic conditions (Costantini et al. 2010, Aroudj and Ouarab 2025). The consistent production of a third clutch following failure of the second clutch suggests that kestrels may maintain reproductive readiness throughout an extended breeding window, likely as an adaptive response to high nest loss rates in natural populations.

Recent evidence indicates that replacement clutching may occur across a broader range of raptor taxa than previously assumed. Replacement clutches have now been documented even in large, long‑lived species traditionally considered single‑brooded, such as the Greater Spotted Eagle (Clanga clanga) and the Chaco Eagle (Buteogallus coronatus), provided that clutch loss occurs early and that food availability is sufficient (Gallego-García and Sarasola 2025, Maciorowski et al. 2026). These findings support the interpretation that re‑nesting capacity in raptors is primarily constrained by ecological conditions rather than strict physiological limits.

### Induced replacement clutching in larger falcons

In Peregrine and Saker Falcons, triple clutching in the present study clearly followed an induced replacement‑clutch mechanism. Removal of eggs after the fourth or fifth egg consistently triggered renewed laying approximately 20 days later, demonstrating a predictable reproductive response to clutch loss. Such protocols have long been employed in raptor propagation, especially in Peregrine Falcons and remain a cornerstone of conservation‑orientated captive breeding programmes (Morrison and Walton 1980, Burnham et al. 2003).

### Reproductive output and species‑specific patterns

Marked differences were evident in reproductive output amongst species. Peregrine Falcons exhibited consistently high fertility and chick survival across all clutches and seasons, in agreement with previous studies reporting high reproductive performance of this species under stable captive conditions (Burnham et al. 2003). Saker Falcons also showed high fertilisation success, although a gradual reduction in egg size was observed in later clutches. A similar tendency was observed in Peregrine Falcons, particularly in the third clutch and after repeated laying within the same breeding season. Progressive egg size reduction after the eighth to tenth egg laid within a season has been reported in other raptors and is generally interpreted as a consequence of cumulative energetic costs of sustained egg production (Durant et al. 2004, Costantini et al. 2010). As egg size represents an important component of reproductive investment, reductions in egg dimensions may reflect changes in female condition, food/nutrient availability or the cumulative cost of repeated egg formation (Wiebe and Bortolotti 2009, Watson et al. 2015). Importantly, reduced egg size did not impair hatchability or chick survival in the present study, consistent with findings from long‑term captive breeding programmes in Saker Falcons (Petrov et al. 2021, Arkumarev et al. 2025). This suggests that, under the husbandry conditions applied here, smaller egg size in later clutches represented a measurable effect of repeated reproductive effort rather than an immediate reduction in breeding success.

In Common Kestrels, reproductive success was constrained primarily by external factors rather than intrinsic physiological limitations. Predation accounted for the complete loss of second clutches; nevertheless, females rapidly resumed laying and produced viable third clutches. Similar reproductive resilience has been reported in wild kestrel populations experiencing high nest failure rates (Aroudj and Ouarab 2025).

### Physiological limits and welfare considerations

Although the present results demonstrate considerable reproductive capacity, repeated clutch production is likely associated with physiological costs that may not be immediately apparent. Experimental and observational studies have shown that sustained egg production can influence adult condition, egg volume and future reproductive performance (Durant et al. 2004, Costantini et al. 2010). The observed reduction in egg size during later clutches may, therefore, be interpreted as a potential indicator of cumulative reproductive strain. Repeated egg formation requires continued allocation of protein, lipids, calcium, vitamins and other micronutrients and partial depletion of these reserves may reduce the resources available for investment in each subsequent egg.

Hormonal and reproductive mechanisms may also contribute to this pattern. In induced replacement clutching, egg removal stimulates renewed laying, but repeated activation of the reproductive cycle within a short period may alter the balance between continued egg production and investment per egg. Smaller eggs in later clutches may, therefore, reflect either physiological limitation or an adaptive adjustment in reproductive investment, allowing females to continue producing viable eggs while reducing the energetic cost of each egg. Recent studies have also documented that alternative or flexible breeding strategies may occur more frequently than previously assumed when energetic and environmental constraints are reduced, even in typically single‑brooded raptors (Rosenfield et al. 2024). While induced clutching can substantially increase short‑term productivity, its long‑term effects on breeding females warrant careful monitoring, particularly in conservation contexts. For this reason, egg size should be monitored as a practical welfare and management indicator during induced multi‑clutch breeding programmes, especially when repeated laying is stimulated through systematic egg removal.

### Implications for conservation breeding

The findings have direct implications for conservation‑orientated captive breeding. In species such as Peregrine Falcons and Common Kestrels, triple clutching can markedly increase the number of juveniles available for release within a single breeding season. In Saker Falcons, where captive breeding contributes to both conservation recovery and regulated use, induced multi‑clutching represents an effective strategy for increasing productivity of genetically valuable breeding pairs (Petrov et al. 2021, Petrov and Dicheva 2024). Comparable management‑driven increases in reproductive output have also been reported in other raptor conservation programmes that applied targeted reproductive manipulation and supplementary management to maximise productivity (Ferrer et al. 2017).

The occurrence of natural triple clutching in Common Kestrels suggests that, in some species, management strategies focused on environmental conditions and food availability may enhance reproductive output without direct intervention. In contrast, larger falcons appear to require deliberate reproductive manipulation to achieve comparable productivity.

### Limitations and future directions

The principal limitation of this study is the small number of breeding pairs per species. Additionally, all observations were conducted under captive conditions with predictable food supply and limited disturbance, representing optimal rather than typical circumstances. Consequently, the results should be interpreted as demonstrating reproductive potential rather than average performance expected in wild populations. Future studies should focus on long‑term monitoring of repeatedly breeding females and comparative analyses between captive and wild populations to better understand the ecological and physiological limits of multiple clutching in falcons.

## Conclusions

This study demonstrates that triple clutching within a single breeding season is biologically possible in falcons under favourable captive conditions. In all three investigated species — Common Kestrel, Saker Falcon and Peregrine Falcon — both monitored breeding pairs successfully produced three consecutive clutches within one season, although the underlying mechanisms differed amongst species. Triple clutching occurred naturally in Common Kestrels under full parental care, whereas in Saker and Peregrine Falcons, it resulted from induced replacement clutching following systematic egg removal.

The findings highlight a high degree of reproductive plasticity in falcons and demonstrate that sustained egg production can be achieved when energetic constraints are minimised and breeding conditions are carefully managed. While induced multi‑clutching substantially increased short‑term reproductive output in larger falcons, its application should be accompanied by close monitoring of adult condition to ensure long‑term welfare and breeding viability.

From a conservation perspective, the ability to obtain multiple clutches from a single breeding pair within one season can significantly enhance the efficiency of captive breeding programmes, particularly for species involved in re-introduction or population reinforcement efforts. Nevertheless, given the small number of breeding pairs examined and the artificial nature of captive conditions, these results should be interpreted as demonstrating reproductive potential rather than typical performance in the wild. Further long‑term studies integrating physiological, behavioural and welfare assessments are needed to define safe management thresholds for repeated breeding in falcons.

## Figures and Tables

**Table 1.** Falcon feeding protocol.

Feeding component / management step	Frequency / timing	Purpose / note
Rodents	3 times per week (Weeks 1 and 2)	Main component of the diet; whole-prey item providing a balanced source of protein, fat, bones and internal organs
Rabbits	2 times during Week 1; 1 time during Week 2	Additional whole-prey source contributing dietary variety
Day-old chicks	1 time per week	Nutrient-rich prey item containing residual yolk reserves that provide readily available energy, lipids, vitamins and micronutrients
Chicken hearts	1 time during Week 2	Protein-rich food item included to increase dietary protein intake and dietary diversity
Fasting day	Sunday (both weeks)	Implemented to better mimic natural fluctuations in prey availability
Post-hatching feeding regime	From hatching of the first chick	Fasting days discontinued and rodent provision increased to meet the elevated energetic demands of breeding adults and growing chicks
Prey condition	Throughout the study	Only freshly euthanised or freshly frozen prey was provided
Vitamin and mineral supplementation	Throughout the breeding season	Versele-Laga MutaVit, Versele-Laga Opti-Breed, Versele-Laga Calci-Lux and Masalles Falcon Top
Additional breeding-season supplements	At the onset of the breeding season	Masalles Chick Complex and Masalles Probiotic
Feeding time	Warmer hours of the day	Intended to better match natural hunting and feeding activity patterns

**Table 2.** Two-week feeding rotation.

**Week 1**	**Week 2**
Rodents × 3 Rabbits × 2 Day-old chicks × 1 Fasting day × 1	Rodents × 3 Rabbits × 1 Day-old chicks × 1 Chicken hearts × 1 Fasting day × 1
6 feeding days + 1 fasting day	6 feeding days + 1 fasting day
**Week 1 after hatching**	**Week 2 after hatching**
Rodents × 4 Rabbits × 2 Day-old chicks × 1	Rodents × 4 Rabbits × 1 Day-old chicks × 1 Chicken hearts × 1
7 feeding days	7 feeding days

**Table 3.** Common Kestrel output.

First pair Common Kestrels	Laid eggs	Fertilised eggs	Successfully hatched chicks	Fledged juveniles	Second pair common kestrels	Laid eggs	Fertilised eggs	Successfully hatched chicks	Fledged juveniles
First clutch 2011	6	6	5	5	First clutch 2011	6	6	5	5
Second clutch 2011	5	rat predation	0	0	Second clutch 2011	5	rat predation	0	0
Third clutch 2011	4	4	4	4	Third clutch 2011	5	5	4	4
First clutch 2012	6	rat predation	0	0	First clutch 2012	5	rat predation	0	0
Second clutch 2012	6	6	5	5	Second clutch 2012	5	5	5	5
Third clutch 2012	5	5	4	4	Third clutch 2012	5	5	4	4
**Total**	**32**	**21**	**18**	**18**		**31**	**21**	**18**	**18**

**Table 4.** Summary of Common Kestrel breeding success rate.

Pair	Laid eggs	Fertilised egg	Successfully hatched chicks	Fledged juveniles	Fertility rate (%)	Hatching success (%)	Fledging success (%)
Pair 1	32	21	18	18	65.6	85.7	100.0
Pair 2	31	21	18	18	67.7	85.7	100.0
Total	63	42	36	36	66.7	85.7	100.0

**Table 5.** Saker Falcon output.

First pair Saker Falcon	Laid eggs	Fertilised eggs	Successfully hatched chicks	Fledged juveniles	Second pair Saker Falcon	Laid eggs	Fertilised eggs	Successfully hatched chicks	Fledged juveniles
First clutch 2015	5	5	5	5	First clutch 2015	5	5	5	5
Second clutch 2015	5	5	5	5	Second clutch 2015	5	5	5	5
Third clutch 2015	5	5	5	5	Third clutch 2015	5	5	4	4
First clutch 2016	5	5	5	5	First clutch 2016	5	5	5	5
Second clutch 2016	5	5	5	5	Second clutch 2016	5	5	4	4
Third clutch 2016	5	5	5	5	Third clutch 2016	4	4	4	4
**Total**	**30**	**30**	**30**	**30**		**29**	**29**	**27**	**27**

**Table 6.** Summary of Saker Falcon breeding success rate.

Pair	Laid eggs	Fertilised egg	Successfully hatched chicks	Fledged juveniles	Fertility rate (%)	Hatching success (%)	Fledging success (%)
Pair 1	30	30	30	30	100.0	100.0	100.0
Pair 2	29	29	27	27	100.0	93.1	100.0
Total	59	59	57	57	100.0	96.6	100.0

**Table 7.** Peregrine Falcon output.

First pair Peregrine Falcon	Laid eggs	Fertilised eggs	Successfully hatched chicks	Fledged juveniles	Second pair Peregrine Falcon	Laid eggs	Fertilised eggs	Successfully hatched chicks	Fledged juveniles
First clutch 2019	5	5	5	0	First clutch 2019	5	5	5	0
Second clutch 2019	5	5	4	0	Second clutch 2019	4	4	3	0
Third clutch 2019	4	4	4	0	Third clutch 2019	4	4	4	0
First clutch 2020	5	5	5	0	First clutch 2020	5	5	5	0
Second clutch 2020	4	4	4	0	Second clutch 2020	4	4	3	0
Third clutch 2020	4	4	3	0	Third clutch 2020	4	4	3	0
**Total**	**27**	**27**	**25**	**0**		**26**	**26**	**23**	**0**

**Table 8.** Summary of Peregrine Falcon breeding success rate.

Pair	Laid eggs	Fertilised egg	Successfully hatched chicks	Raised juveniles	Fertility rate (%)	Hatching success (%)	Raising success (%)
Pair 1	27	27	25	25	100.0	92.6	100.0
Pair 2	26	26	23	23	100.0	88.5	100.0
Total	53	53	48	48	100.0	90.6	100.0
